# Developing data interoperability using standards: A wheat community use case

**DOI:** 10.12688/f1000research.12234.2

**Published:** 2017-12-06

**Authors:** Esther Dzale Yeumo, Michael Alaux, Elizabeth Arnaud, Sophie Aubin, Ute Baumann, Patrice Buche, Laurel Cooper, Hanna Ćwiek-Kupczyńska, Robert P. Davey, Richard Allan Fulss, Clement Jonquet, Marie-Angélique Laporte, Pierre Larmande, Cyril Pommier, Vassilis Protonotarios, Carmen Reverte, Rosemary Shrestha, Imma Subirats, Aravind Venkatesan, Alex Whan, Hadi Quesneville

**Affiliations:** 1 INRA, UAR 1266 DIST Délégation Information Scientifique et Technique, Centre de recherche Ile-de-France-Versailles-Grignon, Versailles, 78000 , France; 2 Unité de Recherche Génomique-Info (URGI), INRA, Université Paris-Saclay, Versailles, 78026, France; 3 Bioversity International, Montpellier, 34397, France; 4 School of Agriculture, Food and Wine, University of Adelaide, Glen Osmond, SA, 5064, Australia; 5 Institut National de la Recherche Scientifique, Centre National De La Recherche Scientifique, Montpellier, 34000, France; 6 Department of Botany and Plant Pathology, Oregon State University, Corvallis, OR, 97331, USA; 7 Department of Biometry and Bioinformatics, Institute of Plant Genetics, Polish Academy of Sciences, Poznań, 60-479, Poland; 8 Earlham Institute , Norwich, NR4 7UZ, UK; 9 International Maize and Wheat Improvement Center, Texcoco, 56237, Mexico; 10 Center for Biomedical Informatics Research, Stanford University, Stanford, CA, 94305, USA; 11 Laboratory of Informatics, Robotics and Microelectronics of Montpellier , University of Montpellier, Montpellier, 34090, France; 12 Institut de Biologie Computationnelle, Université Montpellier, Montpellier, 34090, France; 13 Institut de Recherche pour le Développement , Marseille, 13572, France; 14 NEUROPUBLIC S.A., Piraeus, GR18545, Greece; 15 IRTA. Ctra. de Poble Nou, Sant Carles de la Ràpita, E-43540, Spain; 16 Food and Agriculture Organization of the United Nations, Rome, 00153, Italy; 17 Commonwealth Science and Industrial Research Organisation, Agriculture and Food, Canberra, ACT, 2601, Australia

**Keywords:** wheat, data interoperability, metadata, ontology repository, bio-ontologies, standard vocabularies, data formats

## Abstract

In this article, we present a joint effort of the wheat research community, along with data and ontology experts, to develop wheat data interoperability guidelines. Interoperability is the ability of two or more systems and devices to cooperate and exchange data, and interpret that shared information. Interoperability is a growing concern to the wheat scientific community, and agriculture in general, as the need to interpret the deluge of data obtained through high-throughput technologies grows. Agreeing on common data formats, metadata, and vocabulary standards is an important step to obtain the required data interoperability level in order to add value by encouraging data sharing, and subsequently facilitate the extraction of new information from existing and new datasets.

During a period of more than 18 months, the RDA Wheat Data Interoperability Working Group (WDI-WG) surveyed the wheat research community about the use of data standards, then discussed and selected a set of recommendations based on consensual criteria. The recommendations promote standards for data types identified by the wheat research community as the most important for the coming years: nucleotide sequence variants, genome annotations, phenotypes, germplasm data, gene expression experiments, and physical maps. For each of these data types, the guidelines recommend best practices in terms of use of data formats, metadata standards and ontologies. In addition to the best practices, the guidelines provide examples of tools and implementations that are likely to facilitate the adoption of the recommendations.

To maximize the adoption of the recommendations, the WDI-WG used a community-driven approach that involved the wheat research community from the start, took into account their needs and practices, and provided them with a framework to keep the recommendations up to date. We also report this approach’s potential to be generalizable to other (agricultural) domains.

## Introduction

Wheat was one of the first domesticated food crops, and for 8000 years it has been the basic staple food of major civilizations in Europe, West Asia, and North Africa. According to the International Wheat Initiative (WI, http://www.wheatinitiative.org/), a framework to establish strategic research and organization priorities for wheat research at the international level in both developed and developing countries, wheat is the most widely grown cereal grain, cultivated in about 17% of the total arable land globally, and the staple food for 35% of the world’s population, providing 20% of all calories consumed by people worldwide and more protein in the human diet than any other crop ( http://www.wheatinitiative.org./about-wheat/factsheets-infographics). According to the Consultative Group on International Agricultural Research’s research program on Wheat ( http://wheat.org), an estimated 1.2 billion poor people depend on wheat, a crop that is particularly vulnerable to climate change.

The WI has identified easy access and interoperability of all wheat-related data as a top priority for the wheat research community, which is in line with FAIR data principles ^1^. Interoperability is the ability of two or more systems and devices to cooperate and exchange data, and interpret that shared information ^2^. An important goal is to make the best possible use of the existing and upcoming wealth of genetic, genomic, and phenotypic data in fundamental and applied wheat science. Hence, data interoperability has become a hot topic in this community, given the ever-growing data deluge coming from improvements in data generating technologies and large-scale computational methods for handling DNA and RNA sequencing, high throughput genotyping and phenotyping, high throughput imaging, and satellite monitoring. However, achieving data interoperability is difficult not only because of data and tool heterogeneity, i.e., the ‘ *technical debt’*, but also because of social and scientific issues, such as lack of curation experts, lack of value chains for data generators, and lack of a first class digital citizen recognition for data managers, i.e. the ‘ *cultural debt’*.

To help address these debts, the Wheat Data Interoperability Working Group (WDI-WG, https://www.rd-alliance.org/groups/wheat-data-interoperability-wg) was created as one of the Research Data Alliance working groups ( https://www.rd-alliance.org/groups), under the umbrella of the WheatIS Expert Working Group ( http://wheatis.org/), which is endorsed by the WI to build an international information system for wheat genetic, genomic and phenotypic data. The Working Group included wheat scientists, as well as ontologists and data experts from different organizations and countries, and its mission was to provide a common framework for describing and representing data with respect to existing open data standards. From the outset, the objective of the WDI-WG was to deter communities from creating new standards, which would have made the already-complex landscape of existing data standards even more complex. The WDI-WG collected valuable information through two surveys of the wheat research community, comprising responses regarding existing data formats, practices, and the use of ontologies and controlled vocabularies. From these surveys, the WDI-WG then developed a set of specific recommendations, and worked to facilitate data interoperability through the harmonization of data formats, data models and vocabularies usage, thus aiming to address the main interoperability issues. The proposed recommendations have been endorsed by the WheatIS Expert Working Group and the Technical Advisory Board of the RDA (RDA-TAB).

This paper describes the results and the collaborative methodology used by the WDI-WG, which we believe will be of interest to formalize data interoperability in other crop research communities.

## Developing the recommendations

### A community driven methodology

**Figure 1.**
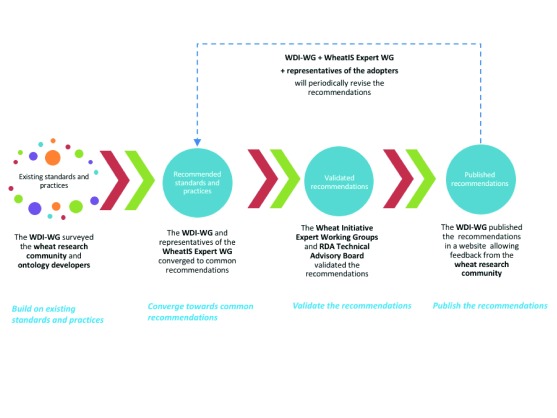
A community driven methodology for data interoperability guidelines design.

From the preparation to the publication of the recommendations, the WDI-WG strongly based its work on the wheat research community. Similarly, the maintenance of the recommendations will be reliant on feedback of the community and the review of a steering group, which includes representatives of the adopters of the guidelines. The main steps of the methodology adopted by the WDI-WG are represented in Figure 1 and are described in more detail in the rest of this section.

### Building on existing standards and practices

The WDI-WG standpoint was to build on prior practices in use in the community, reusing existing standards as much as possible. Gaps, if they existed, could then be filled through the development of new standards. This principle led the working group to start with two surveys, interrogating the wheat research community through the WI communication channels. The first, “Data standards in the wheat research community wheat data interoperability WG”, studied the usage of data standards in the wheat research community through a series of questions sent out to researchers and stakeholders in wheat science. The questions and answers are presented in a report ^3^ and summarized in SuppMat1. The results allowed the group to identify the most commonly used data formats and controlled vocabularies in the wheat research community. The second survey, “Towards a Comprehensive Overview of Ontologies and Vocabularies for Research on Wheat”, focusing on ontologies and vocabularies, allowed the WDI-WG to collect information about the visibility, interoperability, domain, and content of relevant ontologies and vocabularies. The questions and answers of this survey are also presented in a report ^4^ and summarized in SuppMat2.

### Converging towards the recommendations

Two meetings of the WDI-WG were organized in 2014 and 2015, as well as regular face-to-face and online meetings, to analyze the survey results in order to draw recommendations. Calls for participation were regularly posted on the websites of RDA and WI and channeled by the stakeholders. During these working sessions, wheat research scientists, data and information managers, and semantic web experts discussed and collectively agreed on a set of recommendations to cover the widest set of requirements of the communities they supported. The criteria used to guide the recommendation process were the following: (i) reuse existing standards and reinforce existing good practices with regards to interoperability to preserve synergies that work well in the community and (ii) promote emerging standards and practices where gaps exist.

For the following data types of interest to the WDI-WG, the surveys confirmed the existence of adequate consensus regarding data exchange formats: DNA sequence and any associated variants, genome/transcriptome annotations, gene expression data, and physical maps. As such, the WDI-WG recommended the formats that were the most used and/or compliant with the most popular tools and/or already interoperable with other data formats. For example, the GFF3 file format ( http://gmod.org/wiki/GFF3) is found to be widely used by the community to represent genome annotations. Moreover, a Genbank-to-GFF3 script converter is available ( http://www.hpa-bioinformatics.org.uk/biosnippets/snippets/115), in addition to a GFF3 validator tool ( http://genometools.org/cgi-bin/gff3validator.cgi). Thus, the WDI-WG recommended GFF3 for the representation of genome annotations.

However, unlike the aforementioned data types, the wheat data standards survey did not show good consensus for phenotypes and germplasm in terms of data exchange formats and data description practices. For these data types, the WDI-WG collectively agreed to recommend emerging standards, such as (i) Minimum Information About Plant Phenotyping Experiment (MIAPPE) ^5^ and its ISA-TAB implementation ^5^; (ii) the Crop Ontology ^6^, especially the Wheat Trait Ontology for phenotypes ( http://agroportal.lirmm.fr/ontologies/CO_321); and (iii) the FAO-IPGRI Multi-Crop Passport Ontology ( http://agroportal.lirmm.fr/ontologies/CO_020) for germplasm.

### Validation of the recommendations

Prior to their endorsement by RDA, the resulting recommendations have been reviewed by the WheatIS expert working group. As a deliverable of a RDA working group, the recommendations received feedback from the RDA community and validation from the RDA-TAB.

The WDI-WG also used many of the available channels in order to obtain feedback from the wheat research community. In particular, feedback was requested, and was obtained, from communications through the Wheat Initiative’s website, the Food and Agriculture Organization Agricultural Information Management Standards (AIMS) newsletter, and various national and institutional mailing lists.

### Publishing the recommendations

The recommendations are published on the b2share repository ^7^, and a website ( http://datastandards.wheatis.org), which provides the option to submit comments and suggestions. Thus, recommendations can be updated as required by the wheat community, which is of significance since technologies and practices are constantly evolving. Hence, this kind of media allows keeping the guidelines relevant and useful for the wheat research community.

## Disseminating the recommendations

### The Wheat Data Interoperability Guidelines website

**Figure 2.**
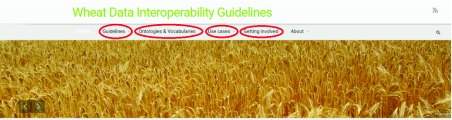
Main items in the menu of the wheat data interoperability guidelines.

**Figure 3.**
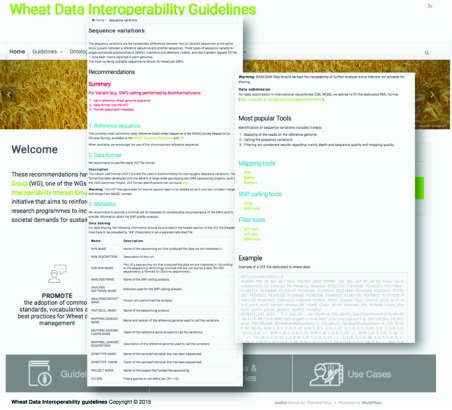
Example of data type specific page (sequence variations).

The first and main output of the WDI-WG is a set of recommendations for describing, representing and linking wheat data. These recommendations are available at http://datastandards.wheatis.org and cover the following data types: sequence variations, genome annotations, phenotypes, physical maps, germplasm, and gene expression. The navigation menu of the website includes four main items ( Figure 2): “Guidelines”, “Ontologies and vocabularies”, “Use cases”, and “Getting involved”. The guidelines menu contains a section for each of the data types addressed by the WDI-WG. Each data type-specific page ( Figure 3) contains the following sections: (i) a summary of the recommendations for the indicated data type; (ii) rationalized recommendations about data format standards; (iii) rationalized recommendations about metadata standards and ontologies; (iv) tools; (v) examples; and (vi) comments. The summary of the recommendations ^7^ and the http://datastandards.wheatis.org website provide detailed information for each data type covered by the guidelines.

In addition to the data type-specific pages, a page dedicated to ontologies and vocabularies explains their benefits and current situation in the context of wheat research data. The use cases page describes examples of use cases with interoperability issues.

### The AgroPortal repository for wheat-related vocabularies

In the context of research data, the use of common vocabularies or ontologies plays a key role in managing, publishing, and reusing data ^8^. Words may have different meanings to different people, and standard definitions for these words are key to avoid miscommunication and to enable good collaboration. Standardized vocabularies and ontologies enhance the efficiency of interoperability and the effectiveness of data exchange, thus facilitating the reuse of data by others, as shown by the Crop Ontology and the Planteome projects ( www.planteome.org) on reference ontologies ^6, 9^. A need to offer a common unique repository of standard vocabularies and ontologies relevant for wheat was identified, and the AgroPortal ( http://agroportal.lirmm.fr/) ^10^, a starting project in 2015, was recognized as suitable solution.

AgroPortal is a collaborative initiative to build a repository of vocabularies and ontologies for agronomy, and related domains (plant sciences, biodiversity, and nutrition). By reusing the National Center for Biomedical Ontologies (NCBO) BioPortal technology ^11^, the portal features ontology hosting, search, versioning, visualization, comment, recommendation, and enables semantic annotation, as well as storing and exploiting ontology alignments, all within a semantic web compliant infrastructure. The AgroPortal specifically pays attention to respect the requirements of the agronomy community in terms of ontology formats (e.g., SKOS, trait dictionaries), or supported features (metadata, annotation). AgroPortal addresses the WDI-WG identified need, while offering a set of interesting features for the ontologies being hosted. Therefore, we have created and maintain an explicit group within AgroPortal and its corresponding slice ( http://wheat.agroportal.lirmm.fr/). Slices are a mechanism supported by the platform to allow users to interact (both via user and application programming interfaces) only with a subset of ontologies in AgroPortal. If browsing the slice, all the portal features will be restricted to a subset, enabling users to focus on their specific use cases. As of today, AgroPortal’s wheat group contains 20 ontologies of the 23 identified by the WDI-WG ^4^. Each ontology has been carefully described (with licenses, authority, availability, etc.), and a new metadata property (omv:endorsedBy) is used to show the ontology’s endorsement by the WDI-WG. The wheat slice in AgroPortal will allow the community to share common meanings of the words they utilize to describe and annotate data, which will in turn make the data more machine-readable and interoperable. Furthermore, the slice will enable wheat-related ontology developers to make their ontologies more visible to the agronomic research community, thus contributing to reduce the proliferation of concurrent ontologies on the Web. This slice has been reported in the WDI-WG guidelines web site (section “Ontologies and vocabularies”), and used as a reference resource to identify and select ontologies related to wheat since then.

## Discussion

### Validation issues

The WDI-WG’s guidelines have been collaboratively built and validated under the umbrella of two authoritative organizations (the WheatIS expert working group and RDA, respectively). The Expert Working Groups of the Wheat Initiative have been instrumental in efficiently interacting with the wheat scientific community in order to take into account the needs from the different fields of biology working on this species. Consequently, the needs and the practices of this community were well-addressed. In addition, the WDI-WG took care to build on existing good practices and preserve prevailing strong synergies in the community, while proposing new standards and practices where relevant. This has been achieved by consulting the wheat research community and experts as frequently as needed. This strategic approach ensures a better adoption of the guidelines by the wheat research community.

Despite this initial strong validation process, the WDI-WG anticipates changes to the recommendations, especially due to an evolving landscape of standards and practices. The blog-like website that hosts the recommendations will facilitate rapid implementation of future changes.

One pitfall the WDI-WG managed to avoid is the quest for immediate comprehensiveness. We deliberated focused on the six data types that were considered most relevant by the wheat research community in the coming years. However, the recommendations can be extended to more data types in future.

### Adoption issues

In order to maximize the adoption of the recommendations, the WDI-WG favors a bottom-up approach rather than enforcing the choice of particular standards. Consequently, to begin with, it is better that individual project initiatives develop their own usage of the proposed recommendations and standards, especially since there are some standards that share common concepts, but address different needs. We prefer the community to adopt at least some standards rather than none. We provide guidelines to facilitate the decision of standards suggesting the most widely adopted ones. By developing the tools required to map/convert from one standard to another, it should be possible to bridge data respecting different standards. The important point is that a standard is used to remove ambiguities in data semantics and representation to enable automated processing. At a later date, when several standards have converged or become widely adopted, it could be possible to enforce their usage. But the time needed to reach this second step will vary between the different fields of biology. 

The WDI-WG will develop training programs to increase the adoption of the guidelines. In fact, the guidelines have already been adopted by a number of stakeholders ( http://ist.blogs.inra.fr/wdi/adopters/). However, these are part of large institutions. This highlights the need to provide tools and training to facilitate the adoption of the guidelines within smaller organizations. Two kinds of training will be developed for two types of audience: data managers with technical skills on data management, and biologists with data knowledge. Another target community of adopters of the WDI-WG’s guidelines is software developers. The adoption of the guidelines by this community is essential to showcase the benefits of data interoperability. Therefore, there is a strong need to raise awareness in this community. 

Finally, reengineering legacy data in accordance with the WDI-WG is an open question. Indeed, it requires from data producers and managers to convert legacy data in recommended data formats or learn how to annotate data with specific vocabularies, which is not trivial for anyone. Depending on the use case and/or the value of the data, it may or may not be worth making such efforts. The use of automated tools for the transformation of data in different formats (where applicable) is expected to minimize the human effort required for such processes.

## Follow up and conclusions

As an RDA working group, the WDI-WG is now in an adoption and maintenance phase. Consequently, the WDI-WG will know focus on dissemination and maintenance activities. A steering group, including representatives of the adopters and the WDI-WG chairs, will drive these activities, taking into account the feedback and contributions of the wheat research community. The action plan of the WDI-WG includes: (i) the promotion of the guidelines via development of information material such as flyers or short videos; and (ii) technical and non-technical training for data managers and scientists, respectively. The WDI-WG will also consolidate the wheat vocabularies group and slice within AgroPortal ( http://wheat.agroportal.lirmm.fr/ontologies). In addition to these projects, it is worth mentioning that the methodology and the results of the WDI-WG have inspired the creation of a rice data interoperability working group within the frame of RDA ( https://www.rd-alliance.org/groups/rice-data-interoperability-wg.html).

The recommendations of the WDI-WG are intended for data producers, data managers, data consumers, and software developers. They constitute a key building block for FAIR data ^1^ sharing infrastructures ( https://www.force11.org/fairprinciples). Indeed, the adoption of the recommendations will facilitate the depositing of data within well recognized repositories in addition to make them easily understandable and reusable.

## Supplementary Material

**Supplementary File 1. Summary of survey “Data standards in the wheat research community wheat data interoperability WG”.**

**Supplementary File 2. Summary of survey “Towards a Comprehensive Overview of Ontologies and Vocabularies for Research on Wheat”.**
