# Supplementary material for: Developing data interoperability using standards: A wheat community use case
Source: F1000Res. 2017 Dec 6;6:1843. Originally published 2017 Oct 16. [Version 2] doi: 10.12688/f1000research.12234.2 (PMC5747345; doi:10.12688/f1000research.12234.2)
Supplement: Supplementary file 1 [file f1000research-6-14407-s0000.tgz › 3af503d2-9c38-4edc-8b05-692003cc3d53.pdf]

Supplementary File 1: Summary of survey “Data standards in the wheat research community wheat data interoperability WG”

The survey was launched from the 7th of April 2014 to the 3rd of June 2014.

Regarding the participants

We received in total 201 answers including 125 complete and 77 incomplete answers. In total, we considered 196 answers (6 were removed corresponding to people who answered twice). The respondents were from more than 17 countries.

62% of the participants answered as individual and 8% answered on behalf of a group. The groups mentioned were: Agricultural Model Intercomparison and Improvement Project (AgMIP), Applied Bioinformatics, CIMMYT Data Management team, CIMMYT Global Wheat Program, Genetic and Biotechnology Lab. UNS, Genetic Resources, Jülich Plant Phenotyping Center JPPC, LEPSE, Phenomics group at ACPFG, SEVEN - UMR GDEC, Soft Wheat Quality Lab, UCD Crop Science, UMT CAPTE Avignon, Wheat Germplasm Bank, CIMMYT

The participants came from different expertise domains, but the majority of them (71%) mentioned breeding as the main domain, 49% mentioned bioinformatics and 44% mentioned agronomy. The majority (55%) of the respondents were researchers.

61% of the respondents were data producers. 73% use data produced by others, among them 31% do it very often. 84 out of 119 respondents were willing to use shared databases to store their data but only 43% of them were already doing so. 48% of the respondents mentioned that their organization has a data management policy or guidelines.

Regarding the data types and formats

In order to start with a limited set of data types, we asked the participants which data types they considered as the most important for the next 5 years. The six data types which came first were phenotypes (98 respondents) SNPs (97 respondents), genomic annotations (83 respondents), germplasm (83 respondents), genetic maps (70 respondents), and physical maps (59 respondents).

We also asked which data types were in use by the participants and the six data types which were the most mentioned were: phenotypes (76 respondents), germplasm (66 respondents), SNPs (49 respondents), gene expression (44 respondents), genetic maps (44 respondents) and genomic annotations (41 respondents).

Regarding the data formats used for SNPs

To the question “which formats do you use for SNPs”, we got 35 answers: the two formats which were the most used by both data producers and data users were BAM/SAM and VCF.

For genomic annotations, 3 data formats were mentioned, Genbank flat file, GFF, EMBL. The 3 of them were quite equally used.

For phenotypes, no standardized data format was found to be importantly in use. Most of the respondents (80) said they were using tabulated formats with no more precision.

For genetic maps, Cmap and GnpMap were the most cited, Cmap being the most used.

For physical maps, FPC and Cmap were mentioned, FPC being the most used.

For germplasm, the most cited formats were MCPD and ABCD. However, 26 respondents of the 40 who answered this question said they were using other formats, including in-house formats.

For gene expression, the two formats which were the most cited were text and GEO.

Regarding the use of vocabularies and ontologies

49% of the respondents said they were using ontologies while 50% said they weren’t. The reasons why the latest don’t use ontologies include lack of awareness and skills. Among the most used ontologies there were many standardized ontologies such as Gene Ontology, Crop Ontology, Plant Ontology, Trait ontology, and Sequence Ontology.

Regarding the use of metadata standards

76% of the respondents said they were not using metadata standards while 23% said they were. The reasons why the participants were not using the metadata standards include the lack of awareness and skills, and the lack of adequate metadata standards. The metadata standards mentioned by the respondents include MAGE, MINSEQE, MIAME, Dublin Core and Darwin Core.
