# Supplementary material for: Developing data interoperability using standards: A wheat community use case
Source: F1000Res. 2017 Dec 6;6:1843. Originally published 2017 Oct 16. [Version 2] doi: 10.12688/f1000research.12234.2 (PMC5747345; doi:10.12688/f1000research.12234.2)
Supplement: Supplementary file 2 [file f1000research-6-14407-s0001.tgz › cb68215c-dbb1-46b0-b145-be3b61e8cbe3.pdf]

Supplementary File 2: Summary of survey “Towards a Comprehensive Overview of Ontologies and Vocabularies for Research on Wheat”

We surveyed the developers, curators and managers of ontologies and vocabularies which could be used for data annotation in the Wheat Research area in order to collect:

- general information: Name and acronym of the ontology or vocabulary, Contact person, URL(s) of the ontology or vocabulary, Who or what organization is supporting the ontology or vocabulary, how regularly the ontology or vocabulary is maintained
- Information about the visibility, usability and interoperability: Is the ontology or vocabulary part of any ontology communities or listing services; what License and/or Copyright is used; relevant publications about the ontology or vocabulary; is your ontology or vocabulary used or implemented in any database/repository; do you interlink and/or map to other ontologies or vocabularies; what is the main purpose of mapping your ontology or vocabulary to other resources; what mapping tools have you used; does the ontology/vocabulary have a term or issue tracker, such as those on SourceForge or GoogleCode; do you offer the terms or concepts translated in different languages; do your web services provide a public API (Application Programming Interface), In which languages or standards is the ontology or vocabulary available; what media types for distribution do you use; is your ontology or vocabulary accessible through web services; is your ontology or vocabulary accessible through a SPARQL Endpoint,
- Information related to the domain and content: What are the knowledge domains described by the ontology or vocabulary; does the ontology/vocabulary use formal relationships.

We received 21 complete answers. The vocabularies and ontologies which participated were: AGROVOC, CAB Thesaurus (CABT), Cell Ontology, Chemical Entities of Biological Interest (ChEBI), Crop Ontology (CO), Environment Ontology (ENVO), Experimental Factor Ontology (EFO), Feature Annotation Location Description Ontology (FALDO), NAL Thesaurus (NALT), Phenotype And Trait Ontology (PATO), Plant Experimental Conditions Ontology (PECO), Plant Ontology (PO), Plant Trait Ontology (TO), Population and Community Ontology (PCO), Protein Ontology (PRO), Sequence Ontology SO), Variation Ontology (VariO), Wheat Ontology INRA, Wheat Plant Anatomy and Development Ontology and Crop Research ontology (CO_121 and CO_715, both are part of Crop Ontology, CO), Wheat trait ontology: Embedded in Crop Ontology (CO_321), and WheatPhenotype

Regarding the visibility, usability and interoperability, 40% of the ontologies didn’t have a license at the moment of the survey, 11 of them do not offer the terms or concepts translated in different languages, and only 8 were accessible through web services, which could be a barrier for their usage. However, only 2 of them were not part of any ontology communities or listing services, 95% of them were used or implemented in any database/repository, 15 of them interlink and/or map to other ontologies or vocabularies.
