# Supplementary material for: Developing data interoperability using standards: A wheat community use case
Source: F1000Res. 2017 Dec 6;6:1843. Originally published 2017 Oct 16. [Version 2] doi: 10.12688/f1000research.12234.2 (PMC5747345; doi:10.12688/f1000research.12234.2)
Supplement: Peer review history

# Peer review: Referee response for version 2

**DOI:** 10.5256/f1000research.14407.r28751

**Published:** 2019-01-29

**Authors:** Ramil Mauleon

**Affiliations:** 1 Strategic Innovation Platform, International Rice Research Institute, Metro Manila, Philippines

Satisfied with the authors' response, good work!

I have read this submission. I believe that I have an appropriate level of expertise to confirm that it is of an acceptable scientific standard.

# Peer review: Referee response for version 2

**DOI:** 10.5256/f1000research.14407.r27995

**Published:** 2017-12-21

**Authors:** Peter McQuilton

**Affiliations:** 1 Oxford e-Research Centre, University of Oxford, Oxford, UK

This is a well structured paper that clearly describes the community consensus building required to ensure data interoperability in the wheat community.

 Given this is the second revision of the manuscript, this work is relatively mature and there is little for me to add in way of suggestions to improve the manuscript. Having said that, the manuscript may benefit from further expansion on how both the recommendation itself and specifically the curation of the ontologies in AgroPortal will be maintained in the future. It may also be interesting to add a section on how this work may be disseminated and made useful to stakeholders outside of the Wheat Interoperability community, given that this work has arisen out of an RDA working group.

I have read this submission. I believe that I have an appropriate level of expertise to confirm that it is of an acceptable scientific standard.

# Peer review: Referee response for version 1

**DOI:** 10.5256/f1000research.13244.r27011

**Published:** 2017-10-30

**Authors:** Ramil Mauleon

**Affiliations:** 1 Strategic Innovation Platform, International Rice Research Institute, Metro Manila, Philippines

The opinion article is well written, and addresses a challenge that plant/crop science researchers face: how to systematically manage data from high throughput technologies (ie. next-gen sequencing, high density genotyping, standardized phenotype measurements/observations)

 The inventory made of data standards dealing with wheat research (with greater focus on genomics/sequences and lesser on phenotype and germplasm, as acknowledged by authors) is very comprehensive and I believe covers what most of the wheat community is using.

 Some minor improvements I see that could be done on the main paper itself is to mention directly some important summaries /findings of the survey results, without having to open the links to the results of the survey. For example, a reader might wish to know how many (or what proportion) of the wheat research institutions surveyed have data standards of what kind (making the paper citable directly and showing the importance of the WDI-WG paper). This is one of the few summaries that could be directly shown. Another is the mention of data standards for genotyping data (especially high density ones), directly in the paper, this is very important data type , I had to open the survey to know more about this.

 Readers who wish to use the recommendations would also likely benefit from having concrete examples of documents that implement the recommendations of the paper directly available (again without having to navigate the external website of the cited resources) within the paper itself. Example, a direct example of snippet of GFF3 for a particular genome annotation would be nice. Can you also mention the most recent resource for wheat genome build, the most authoritative (or most widely used) gene naming of wheat genome (as of writing)? This is very important info this type of data. Another example could be a snippet of a phenotyping experiment result (a field book table, for example), wherein the MIAPPE terms, and the ontology terms tagging the phenotyping data observed/measured appropriate for the experiment can be seen? This gives the reader/researcher ideas on how the data standards/guidelines are used in real-world applications. I went to the http://datastandards.wheatis.org/ externally referred site and I did not easily see a sample dataset that could be used as template. 

 In summary, the paper is already in a very mature and good state, just having some important summaries of the survey directly mentioned and providing examples of applications of the standards in easily accessible sample documents would be a welcome addition.

 Best wishes to the authors!

I have read this submission. I believe that I have an appropriate level of expertise to confirm that it is of an acceptable scientific standard.

# Response

**Published:** 2017-11-23

**Authors:** Hadi Quesneville

**Affiliations:** INRA Versailles, France

Dear Ramil,

 Thank you for this positive review and your useful comments. We have modified the manuscript taking into account your suggestions as follows.

 We added a summary of the survey results as supplementary material to facilitate the reading of our article. In particular to provide the user with the current usage of the standard by the wheat community.

 Concerning examples of documents that implement the recommendations, we consider that this is out of the scope of our paper which describes the methodology we followed to propose standards and guidelines, and not what they are. Because of the nature of the recommendation that would evolve with time, we preferred to implement a website that could be updated according to the community usage as explained in the paper. Writing them in a paper would freeze them and this is contrary to our philosophy. The examples can be found on our guideline website.

 Best regards,
